# Determinants of malaria infection in Dembia district, Northwest Ethiopia: a case-control study

**DOI:** 10.1186/s12889-018-5370-4

**Published:** 2018-04-11

**Authors:** Fisseha Agegnehu, Alemayehu Shimeka, Firnus Berihun, Melkamu Tamir

**Affiliations:** 1 Amhara Regional Health Bureau, Bahir Dar, Ethiopia; 2 Department of Epidemiology and Biostatistics, College of Medicine and Health Sciences, University of Gondar, Gondar, Ethiopia; 3 Clinical Psychology and Counseling Unit, University of Gondar Specialized Hospital, Gondar, Ethiopia; 4 Departement of Human Nutrition, College of Medicine and Health Sciences, University of Gondar, Gondar, Ethiopia

**Keywords:** Malaria, Determinant factors, Case-control study

## Abstract

**Background:**

Despite the progress in reducing malaria infections and related deaths, the disease remains a major global public health problem. The problem is among the top five leading causes of outpatient visits in Dembia district of the northwest Ethiopia. Therefore, this study aimed to assess the determinants of malaria infections in the district.

**Methods:**

An institution-based case-control study was conducted in Dembia district from October to November 2016. Out of the ten health centers in the district, four were randomly selected for the study in which 370 participants (185 cases and 185 controls) were enrolled. Data were collected using a pretested structured questionnaire. Factors associated with malaria infections were determined using logistic regression analysis. Odds ratio with 95% CI was used as a measure of association, and variables with a *p*-value of ≤0.05 were considered as statistically significant.

**Results:**

The median age of all participants was 26 years, while that of cases and controls was 22 and 30 with a range of 1 to 80 and 2 to 71, respectively. In the multivariable logistic regression, over 15 years of age adjusted odds ratio(AOR) and confidence interval (CI) of (AOR = 18; 95% CI: 2.1, 161.5), being male (AOR = 2.2; 95% CI: 1.2, 3.9), outdoor activities at night (AOR = 5.7; 95% CI: 2.5, 12.7), bed net sharing (AOR = 3.9; 95% CI: 2.0, 7.7), and proximity to stagnant water sources (AOR = 2.7; 95% CI: 1.3, 5.4) were independent predictors.

**Conclusion:**

Being in over 15 years of age group, male gender, night time activity, bed net sharing and proximity to stagnant water sources were determinant factors of malaria infection in Dembia district. Additional interventions and strategies which focus on men, outdoor work at night, household net utilization, and nearby stagnant water sources are essential to reduce malaria infections in the area.

## Background

Malaria, which is one of the leading causes of illness and death in the world, is caused by protozoan parasites of the genus plasmodium. About 44% of the world’s population is at risk of malaria infection and over 97 countries are affected by the disease. There are 216 million cases and 445, 000 malaria caused deaths worldwide, and the African Region accounts for about 90% of the cases and deaths. Furthermore, 14 sub-Saharan African countries and India carried 80% of the global malaria burden [1].

Despite the progress gained in reducing malarial morbidity and mortality, the disease remains a major public health problem in many countries of the world. The World Health Organization (WHO) report shows that the number of malaria cases globally fell from the estimated 237 million in 2010 to 216 million in 2016, a decline of 18%. Most of the cases were estimated to have occurred in the African Region (90%), followed by Southeast Asia (7%) and the East Mediterranean Region (2%). Moreover, the number of malarial deaths globally fell from the estimated 446, 000 in 2015 to 445, 000 in 2016 [2].

Malaria is one of the main public health problems in Ethiopia; approximately 75% of the landmass is malaria-endemic, and 65% of the population is at risk of malaria infection [3]. According to the Ethiopian Federal Ministry of Health, malaria accounted for 15% of the reported outpatient visits and nearly 15% of the admissions in the country. Furthermore, malaria is among the ten leading causes of inpatient deaths among children less than 5 years of age [4]. A total of 1,127,241 cases of malaria were reported in Amhara Region; 404,926 and 225,818 of these were in West Gojjam and North Gondar zones, respectively [4].

Dembia is one of the most affected districts in the region. Its proximity to the Lake Tana basin (the largest lake in Ethiopia) and high altitude (2000 m above sea level) are the major factors for the endemicity [5]. In 2016, about 138,842 long lasting insecticidal nets (LLINs) were distributed, and 16 kebeles (lowest administrative units) were sprayed. However, the burden of the disease kept increasing; for instance, in over a 46-week interval, 22,166 malaria cases were reported in 2016 in contrast to 10,415 in 2015 [6].

Malaria prevention and control interventions that included mass distribution of LLINs, the scaled up of indoor residual spraying (IRS), the introduction of rapid diagnostic tests (RDTs) at community levels, and the adoption of artemisinin-based combination therapies (ACTs) have been scaled up since 2005, leading to the reduction of the burden of the disease by about 50%, on average [7].

The national malaria indicator survey demonstrates that in Amhara Region the coverage of LLIN and/or IRS increased from 76.2 in 2007 to 86.3% in 2011 and community knowledge of malaria 26.7% to 48.4% in the same period [8]. Even though written data on malaria cases were limited in the health centers in Dembia district, according to the annual performance report of the FMOH 2017, the total malaria cases treated in the health facilities were 1,820,967 [9].

The burden of malaria remains a major public health problem in Dembia district despite the interventions implemented. Therefore, the aim of this study was to identify the determinants of malaria infections in the district.

## Methods

### Study design and setting

**Fig. 1:**
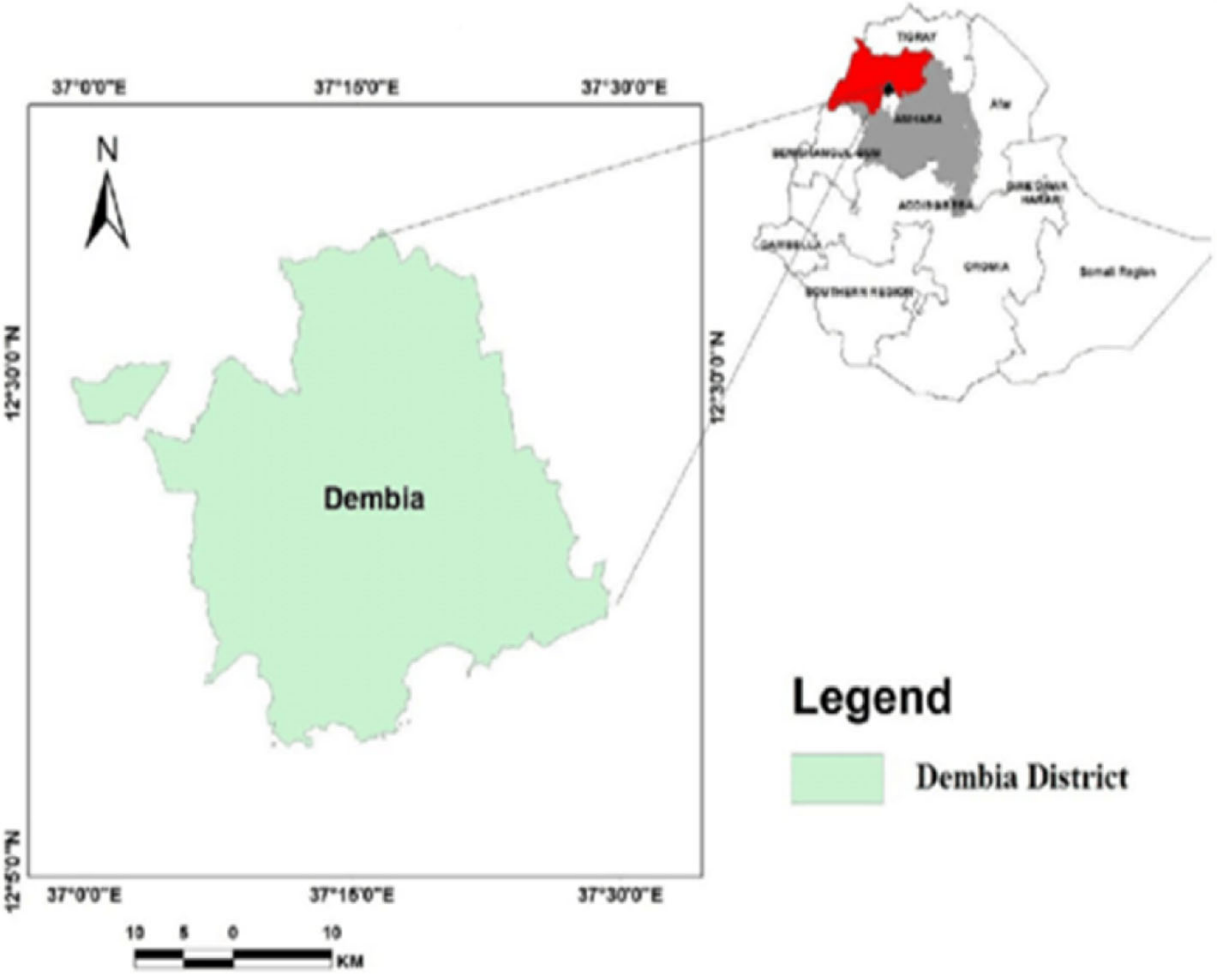
Location of the study area, Dembia district, Northwest Ethiopia (source: Ethio-Geographic Information System (GIS) and (CSA, 2007)

An institution-based matched case-control study was conducted from October to November 2016. The study was conducted in Dembia district, Amhara Region, northwest Ethiopia. It is located in North Gondar zone 729 km north of Addis Ababa at 12°18′30″N and 37°17′30″E. The district has an area of 148,968 sq. km with a total population of 307,967. Out of the total area of the district, plain land accounts for about 87%, mountains and hills 5%, valleys and gorges 4.8%, and water bodies 3.2%. The altitude of the district ranges from 1850 to 2000 m above sea level. The district receives an annual rainfall of 700 to 1160 mm on average [10]. There are 10 health centers and 40 health posts in the district providing healthcare services, such as health education, diagnosis, and treatment (Fig. 1).

The district is divided into 45 sub-districts called Kebeles, the lowest administrative units of Ethiopia. Malaria intervention based on its prevalence is targeted in 16 sub-districts for IRS and the rest for ITN distribution.

**Study design and period:** An institution-based matched case-control study was conducted from October to November 2016.

**Source population:** All individuals living in Dembia district were considered as the source population.

**Study population:** All permanent residents of the districts visiting the health centers.

### Sample size and sampling technique

The sample size for case control study was calculated using the following formula:

$$ \frac{r+1}{r}\frac{\left(p\ast \right)\left(1-p\ast \right)\left( z\beta + za/2\right)2}{\left(p1-p2\right)2} $$

Alternatively, Epi-Info software (matched case-control) method with a p1 (95%), p2 86.3% of ITN/IRS exposure among controls [11], r(1), OR(3), _Zα/2_(95%) and Z1-β(80%) was used to calculate the sample size. Accordingly, n1 was 196 malaria-infected patients, whereas n2 was 196, making a total of 392 participants.

Assuming that all of the health centers were likely to be homogeneous and visited by an equal number of cases, we selected 93 participants each from the four health centers of Dembia district in Ethiopia. We collected data from cases and controls in the four selected health centers for an average of 1 month.

### Case definition

**Cases** were confirmed malaria infected individuals confirmed by microscopy or RDT testes (Care Start®, Access Bio, NJ, USA) at the selected health centers from October 1 to November 10, 2016.

**Controls** were individuals presenting at the same health centers free from malaria infection as diagnosed by microscopy or RDT tests.

### Inclusion criteria

We included permanent residents of the district who visited the health centers and screened for malaria infection using microscopy and RDT tests.

### Exclusion criteria

Participants unable to provide the required information due to different conditions, like extreme illness and absence of caregivers were excluded.

## Study variables

### Dependent variable

The dependent variable was malaria infection among health center attendants.

### Independent variables

**Socio-demographic variables:** gender, age, marital status, educational status, ethnicity, occupation, family size.

**Materials used for malaria prevention related variables:** ITN possession, IRS, number of persons sharing a net.

**Environment-related variables:** housing condition, surrounding water bodies, water source for domestic use.

**Personal activity-related variables:** sleeping outdoors at night, travel history to other malaria endemic areas, sleeping sites.

**Knowledge:** about causes and ways of malaria prevention.

### Data collection procedure

An interviewer-administered questionnaire was employed on infected individuals in the selected health centers in the district to get primary data on demographics and potential risk factors, such as socio-demography, knowledge about malaria, environmental factors, and use of malaria prevention measures. The same questionnaire was used to collect data on potential predictors from the control group. Cases were selected based on definitions developed after they completed their examination (exit interview), and patient cards were immediately taken from the examiner to link the results of the diagnosis. Then, they were interviewed for other relevant bits of information. After the recruitment of cases, controls who lived in the same areas as cases were included.

### Data quality control

The data collection tool was first prepared in English and translated to the local language, Amharic and back translated to English to cheek for consistency. Pre-test was done on 5% of the respondents prior to the actual data collection in a neighboring district. Training was given forboth data collectors and supervisor on the data collection tools and techniques of interviewing with practical exercises. Daily supervision was done by the supervisor and principal investigator.

### Data processing and analysis

Data was cleaned for completeness and consistencies, coded and entered in to Epi info version 7.0 and transported into the Statistical Package for the Social Sciences (SPSS) version 20 for further analysis. The results were organized, summarized and presented using texts, tables, and graphs.

Crude odds ratios (COR) with a 95% confidence interval (CI) were estimated in the bi-variable logistic regression analysis to screen the effect each independent variable on the outcome variable and to select candidate variables for the multi-variable logistic regression analysis. Because of a relatively large number of independent variables considered, we had to screen them using bi-variable analysis to minimize the chance of multicollinearity in the multi-variable regression. Thus, only independent variables with a *p*-value of 0.20 or less in the bi-variable logistic regression were included in the multi-variable logistic regression to get the adjusted effect of each covariate.

Variables which were significant at p-value 0.05 level and 95% CI in the multivariable logistic regression analysis were considered to be the determinant factors of malaria infections.

## Results

### Characteristics of the study population

The median age of all participants was 26 years, while that of cases and controls was 22 and 30 with a range of 1 to 80 and 2 to 71, respectively. The cases group contained more men than women, 74% and 26%, respectively. More than one-third of the participants, 175 (37%), were farmers, that is, agriculture was the main source of income for 98% of the cases and 85% of the controls (Table 1).

**Table 1:** Socio-demographic characteristics of respondents in Dembia district, northwest Ethiopia, 2016 (*N* = 370)

Variable		Case	Control
		*N* = 185(%)	N = 185(%)
Educational status of respondents	No formal education	116(63)	115(63)
	Primary and above	69(37)	67(37)
Educational status of household head	No formal education	165(91)	142(78)
	Primary and above	17(9)	41(22)
Marital status	Married	91(49)	127(69)
	Single	92(50)	56(31)
	Divorced/separated/widowed	2(1)	0(0)
Family size	Mean(range)	5.4(1–12)	5.1(1–12)
Sex	Female	48(26)	100(54)
	Male	137(74)	85(46)
Age	Median(range)	22(1–80)	30(2–71)
Occupation	Farmer	93 (51)	83(45)
	Student	46(26)	24(13)
	Housewife	21(12)	65(35)
	Private	20(11)	33(17)

The majority (85%) of the participants had bed nets, and about one-third of the houses (27%) were treated with IRS in the previous 6 months. Most of the houses (98%) were constructed of mud walls. The sleeping sites of a large proportion (66%) of the respondents were beds without mattresses, and most of (89% cases and 95% controls) the respondents used bed nets consistently (Table 2).

**Table 2:** Housing condition, water source for domestic use, and malaria infection in Dembia district, Northwest Ethiopia, 2016 (*N* = 370)

Variables	Description	Case	Control
		*N* = 185(%)	*N* = 185(%)
Main material of the room’s wall	Mud	182(99)	180(98)
	Wood plunk	2(1)	3(2)
Main material of room’s window	Metallic	9(5)	11(6)
	Wooden	120(65)	127(69)
	No window	55(30)	47(25)
Main material of the room’s floor	Cement	0(0)	2(1)
	Earth	46(25)	36(19)
	Local dung	138(75)	146(80)
Sleeping sites	Bed with mattress	43 (23)	67(36)
	Bed without mattress	136 (74)	110(60)
	Floor	6 (3)	8(4)
Household bed net possession	Yes	152(82)	161(87)
	No	33(18)	24(13)
Indoor residual spraying	Yes	52(28)	47(26)
	No	132(72)	137(74)
Bed net utilization	Yes	137(89)	153(95)
	No	17(11)	8(5)

### Knowledge of participants about causes and preventions of malaria

Cases and controls had comparable knowledge about the causes and preventions of malaria. Most of the respondents (98% of either group) heard about malaria through various means of communication.

Respondents in each group, that is, 87% of the cases and 94% of the controls at least knew that the mosquito was one of the causes of malaria. On the other hand, 84% of the cases and 78% of the controls wrongly held that malaria was acquired either by getting soaked in rain, eating immature sugarcane or contaminated food or by drinking contaminated water. According to 93% of the cases and 94% of the controls, sleeping under any types of bed net was the most common method of preventing malaria (Table 3).

**Table 3:** Knowledge on cause and prevention of malaria infection among residents of Dembia district, northwest Ethiopia, 2016 (*N* = 370)

Variables	Category	Cases	Control
		*N* (%)	*N* (%)
Ever heard of an illness called malaria	No	0(0)	1(0.01)
	Yes	179(98)	182(98)
Cause of malaria	Mosquito	159(87)	173(94)
	Other^a^	153(84)	143(78)
Knew malaria preventability	Don’t know	1(0.5)	0(0)
	No	3(1.6)	0(0)
	Yes	181(98)	182(98)
Prevention ways mentioned	Bed net	169(93)	174(94)
	Avoid mosquito bite	19(10)	25(14)
	Take preventive medication	40(22)	54(29)
	Indoor residual spraying	39(22)	58(31)
	Filling stagnant water	48(27)	48(28)
	Other^b^	90(50)	68(37)

Other^a^-eating immature sugarcane, getting soaked in rain, changing cold weather, eating contaminated food, drinking dirty water

Other^b^-avoiding getting soaked in rain, avoiding eating contaminated food, avoiding drinking dirty water, keeping house clean

### Determinants of malaria infection

In the bivariable logistic regression, gender, age, outdoor activities at night, travel history in the past 2 weeks to other malarious area, habit of sleeping outdoors at night, self reported bed net use, proximity to stagnant water and a temporary river body, and number of persons sharing a bed net were statistically significantly associated with malaria infection at a *p*-value of 0.2. All these variables were included in the multivariable logistic regression model, but other variables which had a *p*-value greater than 0.2 in this model were not included in the multivariable regression model.

When each independent variable was adjusted for other variables, age, gender, sleeping outdoors at night, proximity to stagnant water, and bed net use were found to be statistically significantly associated with malaria infection at a 95% confidence level and a p-value of 0.05.

Age greater than 15 years with an adjusted odds ratio(AOR) and confidence interval (CI) (AOR =18; 95% CI: 2.1–161.5), male (AOR = 2.2; 95%CI: 1.2–3.9), staying outside at night (AOR =5.7; 95% CI: 2.5–12.7), proximity to stagnant water (AOR = 2.7; 95% CI: 1.3–5.4), and three or more people sharing one net had more risk for malaria infection than those sharing one net with two people (AOR = 3.9; 95% CI:2.0–7.7). Variables, such as proximity to temporary river bodies, travel history to another endemic malaria areas, self-reported bed net use, household bed net possession, and household bed net ratio were not statistically significantly associated with malaria infection (Table 4).

**Table 4:** Determinants of malaria infection in Dembia District, northwest Ethiopia, 2016 (*N* = 370)

Variables	Cases	Controls	COR (95 %CI)	AOR (95%CI)
	(*N* = 185)	(*N* = 185)		
Sex				
Male	137	85	3.4(2.2–5.2)	2.2(1.2–3.9)^a^
Female	48	100	1	1
Outdoor activities at night				
Yes	82	31	4.0(2.4–6.4)	5.7(2.5–12.7)^a^
No	103	154	1	1
Travel history in the past 2 weeks to other malaria endemic areas				
Yes	35	11	3.7(1.8–7.5)	1.6(0.5–5.0)
No	150	174	1	1
Sleeping outdoors at night				
Yes	29	8	4.1(1.8–9.3)	0.9(0.2–3.2)
No	156	177	1	1
Bed net use				
Yes	137	153	0.4(0.2–1.0)	0.6(0.2–1.7)
No	17	8	1	1
Household bed net possession				
Yes	152	161	0.7(0.4–1.2)	0.83 (0.8–2.8)
No	33	24	1	1
Proximity to stagnant water body				
Yes	58	34	1.5(1.0–2.4)	2.7(1.3–5.4)^a^
No	127	151	1	1
Proximity to temporary river body				
Yes	127	109	1.5(1.0–2.3)	1.1(0.6–2.0)
No	58	76	1	1
Persons sharing a bed net				
≤ 2	98	51	3.9(2.4–6.2)	3.9(2.0–7.7)^a^
≥3	54	109	1	1
Age (in year)				
≤ 4	10	1	1	1
5–14	37	1	0.3(0.02–4.7)	0.6(0.3–6.2)
≥15	138	183	13.3(1.7–4.8)	18(2.1–161.5)^a^

^a^indicates significant variable using p-value less than 0.05; “1” in Crude odds ratio (COR) and adjusted odds ratio (AOR) indicate the reference category

## Discussion

In this study, being male, 15 and above years of age, staying outdoors at night, bed net sharing with more than 3 persons, and proximity to stagnant water were the determinants of malaria infection. However, ownership and use of ITN were not statistically significant predictors for malaria infection.

In our study, males were more affected by malaria infection than females. This result is in line with those of studies done in Oromia Regional State, Ethiopia, and Eastern Rwanda [12, 13]. This might be so because agriculture is the main occupation and sleeping, and staying outdoors at night is common. Thus, males are more exposed to anopheles mosquito bites. The other possible reason might be that males are particularly less likely to use bed nets even when nets are available [13].

The finding showed that individuals sharing a bed net with more than 3 persons were found to be at more risk for malaria infection than those sharing a net with two other persons. The finding is supported by that of a study done in Laos [14]. In addition, outdoor activity is associated with malarial illness in this study; it is possible that bed nets were not used during outdoors, decreasing their effectiveness in preventing malaria illness. Bed net possession and use is the commonest method of the prevention of malaria infection [9, 15]. In this study, however, it had no statistically significant association with malaria infection contrary to findings of other studies [12, 16]. In this context, these results could derive from a combination of factors, such as the conditions of nets [17], vector resistance issues [18–21], and sleeping sites [22].

Contrary to the findings of other works, IRS was not significantly associated with malaria protection in our study. This may be due to the fact that few households had IRS in the previous 6 months. In addition, it may be due to vector resistance to spray chemicals [18, 23]. Besides, after 5 months, IRS is insufficient to kill resistant mosquitoes [24].

In this study, malaria infection was more widespread among people aged above 15 years. This result is supported by that of a study done in Machinga district, Malawi and WHO malaria report [12, 25]. This might be due to the fact that productive age groups are usually engaged in outdoor activities at dawn and dusk, increase their exposure [13, 26]. Similarly, it is found that outdoor activity at night is one of the significant risk factors for malaria infection in the area. However, this is contrary to the findings in Zimbabwe [26], where individuals below 5 years of age were more at risk of malaria infections. This may be due to cultural differences in that younger individuals are subject to night time outdoor activities in the study area (in Zimbabwe) with no habit of using repellents.

Stagnant water bodies (marshy areas), which are risk factors for malaria infections, are one of the places where malaria vectors breed [27]. Consequently, people near stagnant water bodies are at higher risk for malarial disease than their counterparts.

### Limitation of the study

As a case-control study, our work is prone to selection and recall biases. Besides, LLIN use was reported rather than observed and might have limited the generalizability of the findings.

## Conclusion

Despite the high proportion of bed net possession and self-reported utilization, being male, 15 and above years of age, staying outdoors at night, bed net sharing with more than 3 persons and proximity to stagnant water were the determinants of malaria infection. However, the study identified neither household bed net possession nor self-reported bed net use control measures to be independently associated with malaria infection. Therefore, we focused on men, outdoor activities, and the control of the breeding of the malaria vector on stagnant water bodies, and other bed net distributions to family members in this district to prevent malaria infections.

## Abbreviations

ACT: Artemesinin-based combination therapies
FMOH: Federal Ministry of Health
IRS: Indoor residual spraying
LLNs: Long lasting insecticidal nets
MIS: Malaria indicator survey
RDT: Rapid diagnostics tests
